# Effect of zinc on boar sperm liquid storage

**DOI:** 10.3389/fvets.2023.1107929

**Published:** 2023-02-02

**Authors:** Patricia Marini, Luciana Fernández Beato, Fernando Cane, Juan Manuel Teijeiro

**Affiliations:** ^1^Laboratorio de Medicina Reproductiva, Facultad de Ciencias Bioquímicas y Farmacéuticas, Universidad Nacional de Rosario, Rosario, Argentina; ^2^Consejo de Investigaciones de la Universidad Nacional de Rosario (CIUNR), IBR-CONICET, Rosario, Argentina; ^3^Medax, Santa Fe, Argentina; ^4^Consejo Nacional de Investigaciones Científicas y Técnicas (CONICET), Chañar Ladeado, Argentina

**Keywords:** porcine, spermatozoa, zinc, extender, artificial insemination

## Abstract

Storage and transport of liquid boar sperm for artificial insemination (AI) requires the addition of solutions called extenders, which increase the volume of the ejaculate and help preserve its functional characteristics. Yet, the quality of sperm decreases over time primarily due to the increased production of reactive oxygen species (ROS) that damage the plasma membrane. Many commercial extenders are supplemented with additives that mitigate this effect. In semen, zinc is supplied at high concentration on the seminal plasma and helps protect the plasma membrane of sperm. However, zinc in the seminal plasma is diluted and chelated upon addition of extenders for storage, potentially reducing its antioxidant effect. Here we characterize viability, motility, mitochondrial activity, DNA integrity and ROS content of boar sperm diluted with Sus (Medi Nova, Italy) extender supplemented with different concentrations of ZnCl_2_, at intervals after dilution during 3 days. The ability of sperm supplemented with 2 mM ZnCl_2_ to fertilize oocytes *in vivo* of was also tested. Sperm viability was over 82% for all treatments. Mitochondrial integrity analysis, measured by Cytochrome c activity, indicated a protector effect of Zn, noted as a reduced number of sperm with extensive loss of mitochondrial activity. Acrosomal integrity was improved by treatment with all concentrations of ZnCl_2_ tested. Sperm kinematics were affected by treatment with ZnCl_2_, showing higher percentage of progressive and rapid sperm in doses supplemented with 2mM ZnCl_2_. ROS levels and chromatin integrity did not show differences between ZnCl_2_-supplemented doses and the control. Fertilization rate, total number, live, still born and mummified piglets did not change when sperm were diluted with extender containing 2 mM ZnCl_2_. The presented characterization indicates that Zn addition to Sus extender have a protective effect on mitochondrial sheath and acrosomal membranes; and provides the basis for further studies aimed to optimize sperm performance in AI.

## 1. Introduction

Artificial insemination (AI) in modern pig reproduction requires liquid-stored extended-semen. Extenders provide sperm with nutrients to maintain metabolic processes (1), prevent cold shock (2), control osmotic pressure and pH (3), and antibiotics present in extender inhibit bacterial growth (4). Despite the substantial improvements made on extender formulations in recent years, the quality of stored sperm decreases over time primarily due to the increased production of reactive oxygen species (ROS) during preservation (5). Such oxidative species damage the plasma membrane of the spermatozoa by reacting with the highly abundant polyunsaturated fatty acids at the membrane (6), with subsequent inhibition of respiration and leakage of cellular enzymes (7). Because the integrity of the plasma membrane is essential for sperm interaction with the oviduct epithelium (8) and for the fusion with the oocyte, it is crucial to explore avenues to prevent the detrimental effects of ROS on stored sperm.

In ejaculates, natural antioxidants are provided by the seminal plasma, which is composed of the exocrine secretions of the testicles, the epididymis and male accessory sexual glands (9). Seminal plasma is particularly rich in zinc (10). In sperm, zinc performs numerous functions including regulation of capacitation (11), and regulation of sperm-oviduct and sperm-zona pellucida interactions (12). The concentration of zinc is also particularly high in sperm membranes (13), and its removal by albumin, histidine or EDTA causes membrane defects (14). A large body of evidence indicates that zinc helps maintain functional biological membranes by interacting with enzymes that control their integrity (15). Moreover, zinc has been shown to interact with protein complexes at the membranes, changing their conformation or their substrate specificity, decreasing metal-catalyzed lipid peroxidation (16). Zinc is also an effective scavenger of superoxide anions produced by damaged spermatozoa in human ejaculates (17), and it has been postulated that the high concentration of zinc present in seminal plasma protects the sperm from the detrimental effects of superoxide anions (18). In line with the findings above mentioned, in humans addition of zinc (as zinc sulfate) to ejaculates prior to cryopreservation prevents freeze-thaw-induced DNA damage and helps preserve functional sperm (19). This allows to speculate that adding zinc (as salt) would have benefits to sperm preservation in other species. In boar, liquid seminal doses are prepared after collection of the sperm rich fraction of the ejaculate by dilution in extender and thus components of the seminal plasma, including zinc, are diluted. In addition, most extenders contain EDTA (3) which further depletes zinc from the sperm (20). Even though seminal plasma carries many constituents that are crucial for sperm physiology, because of the proposed roles of zinc as a potent antioxidant we hypothesized that supplementing the extender with zinc could improve the sperm quality upon liquid preservation by reducing the effect of oxidative molecules, and stabilizing the plasma membrane. To test this hypothesis, we extensively characterized the specific effects of zinc added to the extender (without other interfering variables) on viability, motility, mitochondrial activity, DNA integrity and ROS of stored boar semen and its reproductive parameters upon AI.

## 2. Materials and methods

### 2.1. Animals

Boars used in this study were Sire Line PIC 337 and 415 (PIC^®^, Pig Improvement Company, Pasig City, Philippines), Large White (La Botica Genética Porcina S.A., Buenos Aires, Argentina); Pietrain (Cooperativa Marcos Juarez, Cordoba, Argentina); and Landrance (Topigs Norsvin, Buenos Aires, Argentina). A total of 250 seminal doses from 13 boars were used (please, see Supplementary Table 1 for the distribution of seminal doses used). For AI, 250 sows Large White/Landrace, with average parity of 2.1 were used. No nulliparous sows were included. Ethical approval of the research involving animals was given by the Comité Institucional para el Cuidado y Uso de Animales de Laboratorio (CICUAL-FCByF), Universidad Nacional de Rosario (File N° 6060/316).

### 2.2. Experimental design

Semen samples were collected from adult fertile boars by the glove-hand method by seminal doses producer company, Medax (Chañar Ladeado, Santa Fe, Argentina). The frequency of collection was every 7–10 days per boar. All the samples conformed to normal seminal parameters for pig, such as motility > 80% (by CASA, please see complete description in the next section), total sperm in the ejaculate determined by dilution and counting in Neubauer chamber > 20 × 10^9^, and morphological defects [by Wells-Awa staining (21)] < 15%. Sperm-rich fractions of ejaculates were diluted 20 to 30-fold to reach a concentration of 3 × 10^7^ sperm/ml in Sus extender: consisting of monosaccharide energy substrate, ionic salts, Tris (hydroxymethyl) ethane, broad-spectrum antibiotic and 4% EDTA disodium salt (C.A.S. registry 6381-92-6) (Medi Nova, Reggio Emilia, Italy), then aliquots of each seminal dose were supplemented with 0 (control), 0.5, 1, 2, and 3 mM ZnCl_2_ (Merck KGaA, Darmstadt, Germany), and conserved at 16°C until use (up to 3 days). For *in vitro* analyzes, doses were divided into five aliquots and supplemented with ZnCl_2_ in the mentioned concentrations and samples of these aliquots were taken for analysis every 24 h for 3 days. Eight individual doses from eight individual boars were tested for *in vitro* analyzes.

Considering the reported mean value of zinc concentration in seminal plasma of boars is 1,927.5 μg/dL (2.96 mM of Zn^+2^) (22); we used the following final concentrations: 0.5, 1, 2, and 3 mM of ZnCl_2_ to extenders. Doses were maintained at 16°C upon dilution and samples were taken for analysis every 24 h for 3 days.

### 2.3. Sperm viability, acrosome integrity, and motility

Viability (*n* = 8, eight individual seminal doses from eight different boars) was measured using the eosin exclusion test (23). Briefly, 5 μl of sperm suspension were mixed with 5 μl of eosin-nigrosin solution and smeared in a slide.

Acrosome integrity (*n* = 8, eight individual seminal doses from eight different boars) was evaluated by Wells-Awa staining according to characterizations for acrosome alterations in previous works (24). Sperm were observed at magnification of 100× by bright field microscopy. Acrosome intact sperm were considered to be those having a thickened blue-green region at the apex of the head, a blue-green cap covering the anterior two-thirds of the head and a pink color in the posterior one-third of the head. Cytoplasmic droplets and flagellum morphology were also observed. For viability, morphological defects and acrosome integrity analyses at least 200 sperm were counted.

Sperm trajectories were examined using computer-assisted semen analysis (CASA) system (IVOS I Sperm Analyzer, Hamilton Thorne) (*n* = 8, eight individual seminal doses from eight different boars). Aliquots of 8 μl of seminal doses containing 3 × 10^7^ sperm/ml were placed between slides and 18 × 18 mm coverslips. As variations using slides and coverslips instead of commercial chambers are expected, we compared Hamilton Thorne system and the Proiser using ISAS^®^ D4C20 chamber (Supplementary Table 2). No differences were founded between the two methods. Thirty frames were acquired at 60 Hz. The software settings for the IVOS I were: minimum contrast 18, minimum cell size (pix) 7, cell size (pix) 9, cell intensity 125, slow-static cells with average path velocity (VAP) cut-off (l m/s) 20, and straight-line velocity (VSL) cut-off (l m/s) 5, minimum static intensity gates 0.5, maximum static intensity gates 2.5, minimum static size gates 0.65, maximum static size gates 2.6, minimum elongation gates 20 and maximum elongation gates 85 according to Vyt et al. (25). The following parameters of sperm motility were measured: mean path velocity (VAP, μm/s), curvilinear velocity (VCL, μm/s), straight-line velocity (VSL, μm/s), linearity (LIN, %), amplitude of lateral head displacement (ALH, μm), and straightness (STR, %). Sperm with velocities < 15 μm/s. were considered slow, between 16 and 35 μm/s were understood as medium speed sperm and those with a velocity >35 μm/s were considered rapid sperm.

### 2.4. Mitochondria and DNA integrity

Mitochondria and DNA integrity were evaluated simultaneously on the same samples (*n* = 8, 8 individual seminal doses from 8 different boars) by analysis of cytochrome oxidase C activity (26) and sperm chromatin dispersion (27), respectively. Briefly, 100 μl of seminal doses containing 3 × 10^6^ spermatozoa were incubated for 30 min at 37°C with 100 μl of extender solution containing 100 μg of 3,3'-diaminobenzidine (DAB, D-5637 Sigma, Buenos Aires, Argentina). After incubation, 30 μl of suspension were mixed with 70 μl of pre-warmed 1% low-melting agarose at 37°C. Fifteen microliters of suspension were pipetted onto slides pre-coated with 0.65% agarose pre-cooled at 4°C, and covered. The preparation was incubated for 8 min at 4°C, coverslips were removed and sperm incubated for 8 min with acid lysis solution (0.084 N HCl) at 22°C. After washing out the acid lysis solution, samples were incubated for 25 min with lysis solution (0.4 M Tris–HCl pH 7.5, 2 M NaCl, 1% SDS, 0.05 M EDTA, 5% β-mercaptoethanol) at 22°C. Slides were washed for 5 min, dehydrated in sequential 70, 90 and 100% ethanol baths, and stained with Wright solution for 15 min with gentle agitation. Samples were observed using an Olympus microscope BX40 (Japan) at 400× magnification. For evaluation of mitochondria integrity, spermatozoa were classified according to Fariello et al. (28) as follows: pattern I (100% of the midpiece stained), pattern II (>50% of the midpiece stained), pattern III (<50% of the midpiece stained), and pattern IV (no staining of the midpiece) (Supplementary Figure 1A). According to this classification, sperm with pattern I maintained complete mitochondrial activity; with pattern II, lose some mitochondrial activity without impairing cellular motility and fertilizing capacity; with pattern III, presented extensive loss of mitochondrial activity; and with pattern IV were either dead or had minimal energy production. To test DNA fragmentation, we looked at the halos of diffusion of 200 of Wright-stained spermatozoa. Cells with large, medium or small halos of diffusion corresponded to sperm nuclei containing fragmented DNA. Sperm nuclei without DNA fragmentation did not show halos. As control for DNA damage, we incubated aliquots of sperm during 1, 2, and 3 days with 300 μM of H_2_O_2_, but we did not observe effects as described for human sperm (29). Instead, we had to store sperm at−80°C in order to produce damage to the DNA (Supplementary Figure 1B). At least 200 individual sperm were counted.

### 2.5. ROS determination

ROS determination was performed according to Benjamin et al. (30) (*n* = 8, eight individual seminal doses from eight different boars). This method measured a variety of intra and extra-cellular ROS, including ${\text{O}}_{2}^{•-}$, H_2_O_2_, and OH^•^. The results were expressed as relative light units (RLU) per 1.2 × 10^6^ sperm. Cells supplemented with H_2_O_2_ were used as positive control for oxidative damage.

### 2.6. Artificial insemination

Artificial insemination was performed as previously reported (31) in a local farm located in Monte Maíz, Córdoba, Argentina (GPS coordinates: −33.206561, −62.600330). In order to have an approach beyond laboratory conditions and not to skew the observations, we set up the working conditions with the less modifications possible to farm daily operations. Therefore, in our work we tried to explore the effect of zinc in a consuetudinary context instead of laboratory conditions. The AI were performed by day 3 of storage in order to have information from laboratory examinations. Thus, viability, acrosomal integrity and motility were assessed before AI. As neither of these parameters were compromised, then AI was carried out. Personnel who performed AI didn't know about the seminal doses with zinc added. Lactation in the farm is 21-days length. The trial was conducted between November 15th and July 20th 2017. Briefly, estrus was checked daily in the presence of a mature teaser boar. Occurrence of estrus was defined by the standing reflex in front of a boar and back pressure-test. Inseminations were done using spiral catheters (Magapor^®^, Zaragoza, Spain) and 3 × 10^9^ sperm in 100 mL doses were deposited in sow's cervix. All sows were inseminated in standing heat in the presence of a boar. Eighty-six sows chosen at random were allocated to be inseminated with doses supplemented with 2 mM ZnCl_2_ and 164 were inseminated with untreated doses as control. Seminal doses from 13 boars were used. Each individual ejaculate was divided in parts; one part was diluted with extender supplemented with 2 mM ZnCl_2_ and the other with extender without supplementation, and both were used for AI. The distribution of ejaculates per boar used in AI is presented in Supplementary Table 1.

A protector effect on mitochondria integrity and a higher percentage of progressive and rapid sperm in doses supplemented with 2 mM ZnCl_2_ was observed. Also, the percentage of straightness and linearity was higher in doses supplemented with 2 mM ZnCl2 by day 3 of storage. Considering that the kinematic parameters mentioned are a key factor for AI, we performed AI with seminal doses supplemented with 2 mM ZnCl_2_.

### 2.7. Statistical analyses

For viability, motility, acrosome and DNA integrity, and ROS levels, data were analyzed for normality by Shapiro-Wilks's test (32) and Bartlett's test to homogeneity of variances. Then, one-way analysis of variance (ANOVA) for normally distributed data was applied. When F-test results were significant in ANOVA, individual means were further tested by Tukey's multiple range test. Kruskal-Wallis test was applied for data without normal distribution. Assessment of sperm parameters was done using eight biological replicates. Data for progressive motility, VAP, VSL, VCL, ALH, BFC, STR, and acrosome integrity did not show normal distribution. Data regarding mitochondria integrity were analyzed by generalized linear mixed models contemplating interaction between days and treatment. To each variable, a mixed general linear model was adjusted for longitudinal data considering fixed effect for time and treatment, and random effect for boars. Through residuals analysis of the model and Goodness-of-Fit test by Bayesian information criterion (BIC) and Akaike information criterion (AIC), variability intra- and inter-individual were determinate. There was no interaction between time and treatment. Also, variance heterogeneity was considered. For AI trial, the farrowing rate was analyzed by logistic regression model and data regarding to numbers of piglets by Poisson regression. The statistical model that includes the boar factor and corrections made for unequal distribution of semen doses was applied. A random effect for the boar was included to account for the variability and correlation produced by the use of different donors.

## 3. Results

### 3.1. Addition of ZnCl_2_ to the extender affects acrosomal integrity and motility of the sperm upon storage

**Figure 1:**
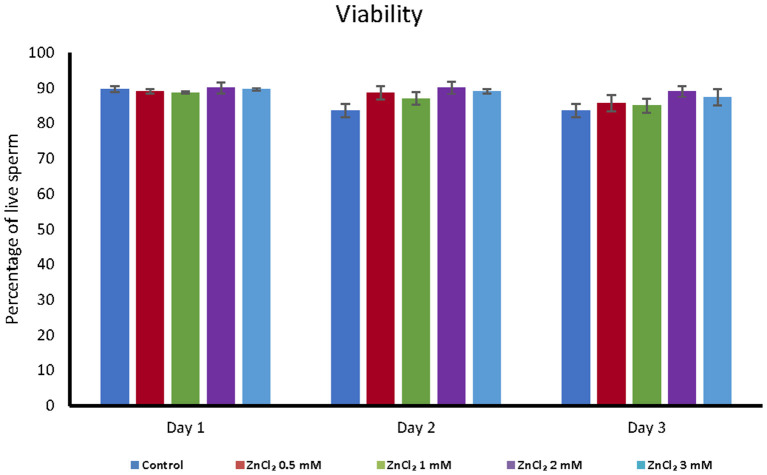
Analyses of viability. Viability was analyzed by eosin-exclusion test in seminal doses supplemented with the indicated amounts of ZnCl_2_ during 1, 2, and 3 days. The results are expressed as mean percentage of live sperm ± standard error (*n* = 8).

**Figure 2:**
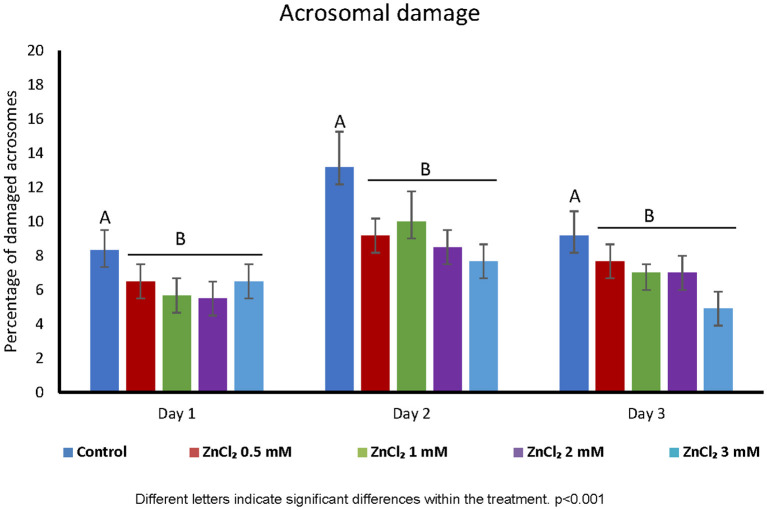
Acrosome integrity. Percentage of damaged acrosomes in sperm diluted with extender containing ZnCl_2_. Acrosomal integrity was evaluated by Wells-Awa technique in seminal doses supplemented with the indicated amounts of ZnCl_2_ during 1, 2, and 3 days. Letters indicate significant differences between treatments. *p* < 0.001; (*n* = 8).

Addition of ZnCl_2_ to the extender did not affect the cell viability based on membrane integrity, regardless of zinc concentration or time elapsed after dilution (Figure 1). However, all doses supplemented with ZnCl_2_ showed a higher percentage of sperm with unaltered acrosomes compared to the control without added zinc (Figure 2).

Table 1 shows the quantification of the sperm motility measured by examination of the trajectories of individual cells. Sperm with slow and medium speeds displayed significant differences between treatments and different kinematic parameters changing through the days in response to added ZnCl_2_. However, the percentage of progressive and rapid sperm in the samples were mainly conserved over time in doses supplemented with 2 mM ZnCl_2_, except for day 3 when a higher percentage of rapid sperm was observed in doses supplemented with 1 mM ZnCl_2_.

**Table 1:** Analyses of motility.

	**Motile (%)**			**Progressives (%)**			**Rap (%)**		
**ZnCl**_2_ **(mM)**	**Day 1**	**Day 2**	**Day 3**	**Day 1**	**Day 2**	**Day 3**	**Day 1**	**Day 2**	**Day 3**
0	89.67 ± 1.45	86.66 ± 2.40	84.66 ± 1.66	29.33 ± 2.40^a^	27.67 ± 1.86^a^	25.33 ± 3.38^a^	60.00 ± 3.06^a^	54.33 ± 2.67^a^	56.00 ± 2.52^a^
0.5	89.33 ± 0.88	88.66 ± 2.30	86.00 ± 1.85	26.67 ± 0.88^a^	32.33 ± 2.03^ab^	29.33 ± 4.48^ab^	53.67 ± 3.48^a^	65.33 ± 6.06^ab^	65.33 ± 2.84^ab^
1	90.33 ± 0.86	85.66 ± 1.76	85.66 ± 1.66	28.00 ± 3.06^a^	29.33 ± 1.76^ab^	33.00 ± 2.65^ab^	56.83 ± 4.33^a^	63.33 ± 3.71^ab^	72.00 ± 4.16^b^
2	92.01 ± 1.10	89.33 ± 0.33	90.66 ± 1.85	40.67 ± 2.06^b^	34.00 ± 1.00^b^	38.67 ± 1.67^b^	74.33 ± 1.20^b^	73.00 ± 3.51^b^	65.00 ± 1.15^ab^
3	90.33 ± 0.33	86.66 ± 0.67	89.66 ± 2.40	30.67 ± 3.28^a^	28.00 ± 2.89^ab^	32.33 ± 2.96^ab^	60.33 ± 0.67^a^	60.67 ± 3.93^a^	60.67 ± 5.17^ab^
	**Med (%)**			**Low (%)**			**LIN (%)**		
**ZnCl**_2_ **(mM)**	**Day 1**	**Day 2**	**Day 3**	**Day 1**	**Day 2**	**Day 3**	**Day 1**	**Day 2**	**Day 3**
0	9.33 ± 0.33^a^	14.33 ± 2.6^b^	17.67 ± 1.45^b^	27.33 ± 2.85^b^	27.33 ± 1.86^b^	23.33 ± 1.20	37.33 ± 2.33	38.00 ± 1.00	34.33 ± 1.86^a^
0.5	13.00 ± 1.53^b^	11.67 ± 1.86^ab^	9.33 ± 1.20^a^	31.00 ± 2.52^b^	21.67 ± 4.06^b^	24.67 ± 4.10	36.67 ± 0.88	37.67 ± 0.67	36.33 ± 3.53^ab^
1	11.17 ± 0.58^ab^	11.33 ± 0.88^ab^	8.33 ± 0.33^a^	29.17 ± 4.70^b^	23.33 ± 3.53^b^	18.33 ± 4.06	37.00 ± 0.00	36.00 ± 1.53	38.67 ± 0.67^ab^
2	8.67 ± 0.67^a^	8.33 ± 1.45^a^	8.33 ± 1.86^a^	15.33 ± 1.86^a^	17.67 ± 2.19^a^	23.00 ± 2.00	40.03 ± 1.98	38.00 ± 1.00	46.67 ± 1.20^c^
3	8.67 ± 1.67^a^	11.00 ± 1.00^ab^	10.00 ± 0.58^a^	29.33 ± 1.20^b^	31.00 ± 4.16^b^	26.67 ± 3.84	39.33 ± 1.86	36.67 ± 1.20	40.33 ± 0.33^b^

Motility was analyzed by CASA in seminal doses supplemented to the indicated concentrations of ZnCl_2_ during 1, 2 and 3 days. Means of percentage of motile, rapid (RAP), medium (MED) and low (LOW) sperm ± standard errors are shown. Linearity (LIN) was also expressed as means of percentage ± standard errors. Different letters indicate significant differences between treatments (n = 8; p < 0.05).

While percentage of motile sperm did not show significant differences between treatments, VAP, VCL, VSL, LIN, ALH, and STR changed after Day 1 (Table 2). By day 2, VAP, VSL and ALH showed the highest values, while BCF showed the lowest values in doses supplemented with 2 and 3 mM of ZnCl_2_. At this time, VCL was higher in treatments at all concentrations of ZnCl_2_. By day 3, VAP, VSL, VCL, showed the higher values at 0.5 and 1 mM of ZnCl_2_, and ALH did not show differences with the control. Zinc-supplemented samples displayed the highest values of LIN at 2 mM ZnCl_2_, and the lowest value of BCF by day 3. These results indicate that addition of ZnCl_2_ to the extender affects the kinetic parameters of the sperm.

**Table 2:** Kinematic parameters of sperm.

	**VAP (**μ**m/s)**			**VSL (**μ**m/s)**			**VCL (**μ**m/s)**		
**ZnCl_2_ (mM)**	**Day 1**	**Day 2**	**Day 3**	**Day 1**	**Day 2**	**Day 3**	**Day 1**	**Day 2**	**Day 3**
0	55.73 ± 4.98	47.23 ± 3.15^a^	53.73 ± 2.29^a^	40.33 ± 3.71^a^	35.20 ± 2.35^a^	38.13 ± 2.15^a^	115.33 ± 9.02	102.63 ± 4.43^a^	117.80 ± 4.91^a^
0.5	54.30 ± 2.43	59.07 ± 3.79^b^	64.37 ± 2.22^b^	39.27 ± 1.56^b^	43.73 ± 2.73^b^	45.97 ± 1.99^b^	114.73 ± 1.55	119.83 ± 6.15^b^	131.17 ± 7.87^b^
1	55.02 ± 3.29	55.30 ± 3.01^ab^	65.97 ± 0.46^b^	34.7 ± 3.08^a^	40.30 ± 2.88^ab^	48.27 ± 0.7^b^	115.03 ± 5.02	116.83 ± 3.47^b^	130.60 ± 2.52^b^
2	61.83 ± 1.93	63.33 ± 1.29^b^	56.40 ± 3.58^a^	47.87 ± 1.73^a^	46.50 ± 1.47^b^	44.60 ± 3.62^ab^	119.83 ± 3.6	128.07 ± 1.95^b^	103.70 ± 4.76^a^
3	56.87 ± 0.88	62.33 ± 1.76^b^	55.57 ± 2.95^a^	42.00 ± 1.88^a^	45.53 ± 1.62^b^	41.93 ± 2.39^ab^	115.90 ± 1.68	126.60 ± 3.13^b^	111.73 ± 5.83^a^
	**ALH (**μ**m)**			**STR (%)**		
**ZnCl**_2_ **(mM)**	**Day 1**	**Day 2**	**Day 3**	**Day 1**	**Day 2**	**Day 3**
0	4.70 ± 0.72	4.33 ± 0.03^a^	5.63 ± 0.34^b^	69.33 ± 1.76	72.67 ± 0.33	68.00 ± 1.73^a^
0.5	5.07 ± 0.18	5.03 ± 0.18^bc^	5.63 ± 0.38^b^	69.00 ± 1.25	70.00 ± 0.58	67.67 ± 3.71^ab^
1	4.88 ± 0.33	4.67 ± 0.12^ab^	5.73 ± 0.28^b^	68.18 ± 0.58	69.00 ± 1.02	69.33 ± 1.2^ab^
2	4.63 ± 0.23	5.33 ± 0.03^c^	3.97 ± 0.12^a^	64.67 ± 1.8	70.00 ± 1.53	75.67 ± 0.33^c^
3	5.87 ± 0.88	4.33 ± 1.76^b^	5.57 ± 2.95^a^	65.90 ± 1.68	69.60 ± 3.13	68.73 ± 5.83^a^

Kinematic parameters observed upon storage with 0, 0.5, 1, 2, and 3 mM of ZnCl_2_ for 1, 2, and 3 days are shown as mean ± standard error. Average path velocity (VAP), straight-line velocity (VSL), curvilinear velocity (VCL), the amplitude of lateral head displacement (ALH) and straightness (STR). Different letters indicate significant differences between treatments (n = 8; p < 0.05).

### 3.2. Added zinc protects mitochondria in stored sperm

**Figure 3:**
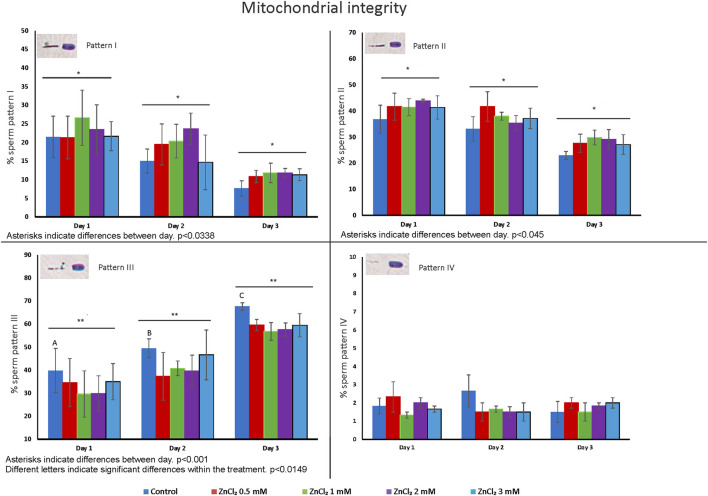
Distribution of cells with staining patterns that report on mitochondria activity. Seminal doses were supplemented with ZnCl_2_ and stored for 1, 2, and 3 days. The graphics show the means of percentage of each pattern of staining of the midpiece based on cytochrome c oxidase activity ± standard error. Asterisks show significant differences between days (*p* = 0.038, *p* = 0.045 and *p* = 0.001 for pattern I, II, and III respectively) and letters show significant differences between treatments (*p* = 0.0149) (*n* = 8). Cells displaying Pattern I maintain full mitochondrial activity. Cells displaying Pattern II have mitochondria without a severe impairment of motility and fertilizing capacity. Spermatozoa with Pattern III have significant loss of mitochondrial activity, and cells with Pattern IV are either dead or have minimal energy production.

Figure 3 shows the percentage of sperm displaying different patterns of staining of the midpiece (I to IV), from 100 % staining (full mitochondrial activity) to no staining (dead cells or cells with no mitochondrial activity) (Supplementary Figure 1A). A decrease in the number of cells showing pattern I and II was observed at all tested times after dilution, with an associated increase of cells with pattern III. But the increase in sperm displaying pattern III (cells that present extensive loss of mitochondrial activity) in the control was significantly higher than those of samples containing added ZnCl_2_ (at all concentrations). An interaction effect between day and treatment was observed for pattern III (*p* = 0.0149). The number of cells displaying pattern IV was not affected by either time or treatment.

**Figure 4:**
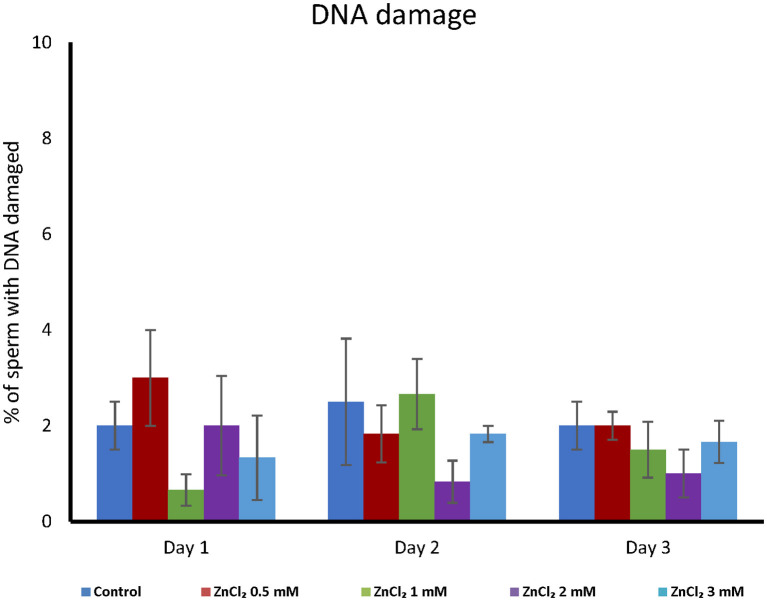
DNA integrity analyses. Seminal doses were supplemented with the indicated concentrations of ZnCl_2_ and analyzed during 1, 2, and 3-days. DNA integrity was measured together with mitochondrial status. The integrity of chromatin did not show differences between treatments or day of evaluation (*n* = 8).

Addition of ZnCl_2_ resulted in no significant differences in DNA fragmentation (see Supplementary Figure 1B), regardless the length of storage (Figure 4).

### 3.3. Zinc does not affect the production of ROS in stored sperm

Table 3 shows the levels of ROS in sperm in the presence and absence of added ZnCl_2_, and over time, expressed as relative units per 1.2 × 10^6^ spermatozoa. Results show that in all tested conditions, the addition of ZnCl_2_ causes no effect on ROS production, at all tested times.

**Table 3:** ROS production.

	**Control**	**ZnCl_2_ 0.5 mM**	**ZnCl_2_ 1 mM**	**ZnCl_2_ 2 mM**	**ZnCl_2_ 3 mM**	**H_2_O_2_ 300 μM**
Day 1	52.69 ± 7.97	47.73 ± 3.04	47.41 ± 1.52	49.71 ± 2.21	47.62 ± 1.55	202.16 ± 58.23^a^
Day 2	46.52 ± 3.35	47.35 ± 5.01	53.42 ± 8.44	46.87 ± 3.41	49.03 ± 3.36	77.83 ± 2.47^b^
Day 3	47.27 ± 5.59	48.33 ± 5.61	48.94 ± 6.17	49.03 ± 6.24	48.33 ± 4.78	94.49 ± 37.78^b^

Seminal doses were diluted with extender containing ZnCl_2_ at the indicated concentrations for 1, 2, and 3 days. ROS levels were evaluated using the Luminol-based technique. Positive control for ROS production contains 300 μM H_2_O_2_. Different letters indicate significant differences between treatments (n = 8; p < 0.05).

The values represent the mean of RLU ± standard error.

### 3.4. Artificial insemination with doses supplemented with 2mM ZnCl_2_

From a total of 250 sows inseminated in the trial, 204 became pregnant. The farrowing rate using doses supplemented with zinc was 85.7%, while the farrowing rate without zinc supplementation was 79.5%. No statistically differences between the control and the ZnCl_2_-supplemented sperm were observed in farrowing rate (Table 4), number and distribution of total number of piglets, live piglets, stillborn and mummified fetuses (Table 5).

**Table 4:** Percentage of pregnant sows upon AI performed using sperm preserved in extender with 2 mM of ZnCl_2_ and without ZnCl_2_.

**Total number of sows used in the trial**			
**Treatment**	**N**°**of sows inseminated**	**N**°**of pregnant sows**	**Farrowing rate**
Without zinc	166	132	79.5%
With zinc	84	72	85.7%

Average farrowing rate of the farm per year: 2015: 77.72%; 2016: 76.84%; 2017: 76.5%; 2018: 75.94%; 2019: 72.98%.

**Table 5:** Data collected from AI performed with sperm stored in the presence and absence of 2 mM ZnCl_2_.

**Distribution of litter size**			
		**With zinc (*****n** =* **1,026)**	**Without zinc (*****n** =* **2,528)**
Total number of piglets	Mean	11.83	10.94
	Standard deviation	2.85	3.27
	Median	12	11
	Interquartile range	4	3
	Minimum	3	2
	Maximum	19	18
Live piglets	Mean	11.24	10.49
	Standard deviation	2.92	3.16
	Median	12	11
	Interquartile range	3	3
	Minimum	0	1
	Maximum	17	18
Stillborn	Mean	0.46	0.37
	Standard deviation	1.10	0.73
	Median	0	0
	Interquartile range	1	1
	Minimum	0	0
	Maximum	10	3
Mummified fetuses	Mean	0.14	0.04
	Standard deviation	0.57	0.20
	Median	0	0
	Interquartile range	0	0
	Minimum	0	0
	Maximum	5	1

The trial was conducted between November 15th and July 20th 2017. No significant differences were found in the reproductive parameters measured (n = 3,554).

## 4. Discussion

Recent studies investigated the role of zinc ion on boar sperm physiology (33). These studies reported the ability of sperm to incorporate zinc from the medium, the redistribution of zinc in the spermatozoon during capacitation, and zinc's role in the regulation of sperm interactions with the oviduct and zona pellucida (11, 12). However, the effects of zinc on liquid boar sperm preservation were not explored. We aimed to analyze improvements on sperm quality by Zn addition upon the best conservation conditions possible. We thought that if improvement is seen even under these conditions, when less quality samples or extenders are used the addition of zinc might become of greater help.

In order to optimize commercial extenders, supplementations with single anti-oxidant compounds were tested. Studies with exogenous antioxidants such as L-glutamine, bovine serum albumin (BSA), skim-milk, taurine, adenosine, L-cysteine hydrochloride, ascorbic acid and magnesium fumarate (34–38) have been reported, but the effect of adding seminal plasma endogenous antioxidant compounds, such as Zn, to the extender has not been tested.

Here, we set to characterize the effect of zinc addition to the extender at different concentrations and over time for 3 days after sperm collection and dilution. By using gloved-hand method some seminal plasma is also collected along with the sperm-rich fraction, this adds more variation in the zinc concentration of each seminal doses produced and restoring the original concentration of zinc in the sperm would require measuring its concentration in each ejaculate, which is technically challenging. Therefore, the concentrations of zinc used in this study were chosen considering that: (1) the concentration of zinc in seminal plasma is usually reported as the mean of a group of animals. Therefore, a single ejaculate may have much higher or lower amounts of zinc than the reported mean; (2) zinc ion is added as ZnCl_2_, and an excess can affect the osmolality of the media and result in cell damage. It is to note that Sus extender contains EDTA, which chelate zinc. As EDTA is in excess to guarantee that all calcium ion were chelated, this affects the zinc availability for spermatozoa. Although the exact concentration of zinc and EDTA present in each seminal doses is unknow, the effects of zinc can be contrasted with appropriated controls. The tested concentrations did not have an adverse effect on viability after 3 days of storage, and they were used to further characterize the stored sperm.

Supplementing the extender with ZnCl_2_ showed a protective effect on acrosome stability (Figure 2) and this is important because acrosome stability has been associated to higher litter size (39). Membrane damage upon sperm preservation is often linked to oxidation of lipids due to the generation of ROS. ROS play a variety of roles in sperm physiology, from spermatogenesis to motility (40) and Zinc is involved in the regulation of ROS levels and is a co-factor for important enzymes involved in the antioxidant defense system (41). Despite these proposed functions as a ROS scavenger, in our study we did not corroborate that Zn acts as an antioxidant during liquid sperm storage (Table 3). This may be due to the presence of seminal plasma in extended semen that acts as ROS scavenger (42). In accordance with this, chromatin integrity was not affected by storage. Thus, as chromatin was stable under the control condition, the zinc stabilizing effect on chromatin previously described (43) could not be observed in our experiments. This is in coincidence with the delay or even abolishment of boar sperm DNA fragmentation in extended boar semen (44). Interestingly, H_2_O_2_ 300 μM is enough to produce damage to DNA in human sperm (29). However, when we use H_2_O_2_ 300 μM as positive control in ROS levels measurements; we did not observe DNA fragmentation in boar extended-sperm (data not shown). Instead, we had to use low temperature (-80°C) to induced damage and validate our DNA fragmentation detection technique (Supplementary Figure 1B).

As ROS concentration did not vary upon treatment, the acrosome stabilizing effect can hardly be attributed to the antioxidant property of zinc. Cellular membranes have been previously shown to be stabilized by zinc (16), and this effect may be responsible for the better preservation of acrosome membranes observed in samples supplemented with zinc. Moreover, an interesting correlation between greater liver zinc concentrations with less acrosomal damage were found in bull (45). The particular composition of the membrane overlying the acrosome (46) may render it more sensitive to damage during storage, thus benefiting from the stabilizing effect of zinc. Also, zinc efflux from sperm activates matrix metalloproteinase 2 (MMP2) located at the inner acrosomal membrane, and zinc is known to decrease the proteolytic activity of the 26S proteasome and MMP2 (12). This effect could also contribute to the increase in acrosomal stability observed in the presence of zinc.

As Zn addition showed effects on preserved sperm motility and this is closely related to mitochondrial function, we evaluated mitochondrial status in sperm preserved with Zn. Zinc exerted a protective effect on mitochondria, as we observed fewer sperm showing pattern III—with extensive loss of mitochondrial activity—on doses supplemented with zinc at all tested concentrations (Figure 3). This is in accordance with a previously reported stabilizing effect of zinc on mitochondrial sheaths (47) and may have a correlation with the changes in kinematics parameters observed. It is interesting to note that mitochondrial as well as peri-acrosomal membranes appear to be stabilized by Zn addition to extenders.

The finding that ZnCl_2_ affects sperm kinematic parameters during preservation is meaningful as different kinematic parameters affect outcomes in fertility performance (39, 48). Ejaculates diluted with 2 mM ZnCl_2_-supplemented extender show the highest percentage of progressive sperm (Table 1) and such kinematic parameter correlates positively with farrowing rate (49). Moreover, addition of 2 mM ZnCl_2_ shows a higher population of rapid sperm until day 2 that maintain straightness in trajectories and linearity by day 3 (Table 2), parameters that correlate positively with total number of piglets born (49, 50). Therefore, those kinematic parameters affected by 2 mM ZnCl_2_ are considered desirable for AI procedures and were taken into account to perform the further AI trial.

To test the possibility that the presence of Zn in Sus extenders affects reproductive outcome, sows were inseminated with sperm preserved in extenders supplemented with 2 mM ZnCl_2_,—the concentration that showed the best progressive and rapid motility. In this proof-of-principle research, in which the number of samples is low, we found no statistical differences in porcine reproductive parameters. However, it is to note that some parameters would potentially improve with zinc treatment and deserve to be studied in the future in a field trial. Considering a small farm, as in this study, with 250 sow and a farrowing rate of 79.5%, there is 2.02 birth per sow/year (contemplating 115 gestation days + 21 weaning days + 7 days to return to estrus cycle). If the total number of piglets would improve in 0.89 for zinc treatment, as in this trial (Table 5). Total number of piglets: 11.83 with zinc – 10.94 without zinc = 0.89); then, 449 more piglets would be gained in the farm (250 × 2.02 × 0.89 = 449.45). Moreover, if we considered a potential increase in farrowing rate (85.7%), then, the birth per sow/year would be 2.18 and there would be 485.05 more piglets (250 × 2.18 × 0.89 = 485.05). This would produce a profit in pork production that deserves to be further studied for AI procedures.

The beneficial effect of zinc addition to Sus extender on some sperm motility parameters, acrosome stability and mitochondria integrity without notable effects on viability, ROS production and AI outcome may result from the combination of several effects, as direct interaction of this element with enzymes and proteins associated to membranes or to other membrane components. The stabilization of the sensitive acrosomal membranes and mitochondria; and the improvement in desirable kinematic parameters by added zinc, emerge as attractive targets for further studies to optimize AI techniques. However, it is to note that the study was carried out with high genetic merit boars which show mainly good quality of sperm. It would be interesting to analyze this effect in boars with low quality of sperm and to perform a new IA trial.

In whole, zinc addition to Sus extender improves some motility parameters and the stability of the acrosomal membrane as well as mitochondria. Later studies are needed to analyze improvement of AI.

## Data availability statement

The original contributions presented in the study are included in the article/Supplementary material, further inquiries can be directed to the corresponding author.

## Ethics statement

Ethical approval of the research involving animals was given by the Comité Institucional para el Cuidado y Uso de Animales de Laboratorio (CICUAL-FCByF), Universidad Nacional de Rosario (File N° 6060/316).

## Author contributions

PM conceived and designed the study and wrote the draft paper. LF performed mitochondrial and DNA integrity test. FC performed artificial insemination. JT conceived and designed the study, performed statistical analysis, analyzed data, wrote the paper, and revised the article for intellectual content. All authors contributed to the article and approved the submitted version.

## Supplementary material

Supplementary Figure 1 Patterns of mid-piece staining and DNA integrity. **(A)** Microscope images of sperm showing different patterns of staining of the midpiece based on cytochrome c oxidase activity: pattern I, 100% of the midpiece stained, pattern II, more than 50% of the midpiece stained, pattern III, less than 50% of the midpiece stained, and pattern IV, no staining. **(B)** The brown color is product of DAB precipitation. **(B)** Micrograph showing different degrees of DNA damage based on Wright solution staining. Cells with large, medium or small halos of diffusion correspond to sperm nuclei containing fragmented DNA. Severe DNA damage was obtained by storing sperm at −80°C. Different grades of damage are seen on the micrograph **(A)**.

Supplementary Table 1 Distribution and origin of boars used for AI trial.

Supplementary Table 2 Comparison between two CASA systems with two different supports.
