# Supplementary figures and images for: Effect of zinc on boar sperm liquid storage
Source: Front Vet Sci. 2023 Feb 2;10:1107929. doi: 10.3389/fvets.2023.1107929 (PMC9932539; doi:10.3389/fvets.2023.1107929)

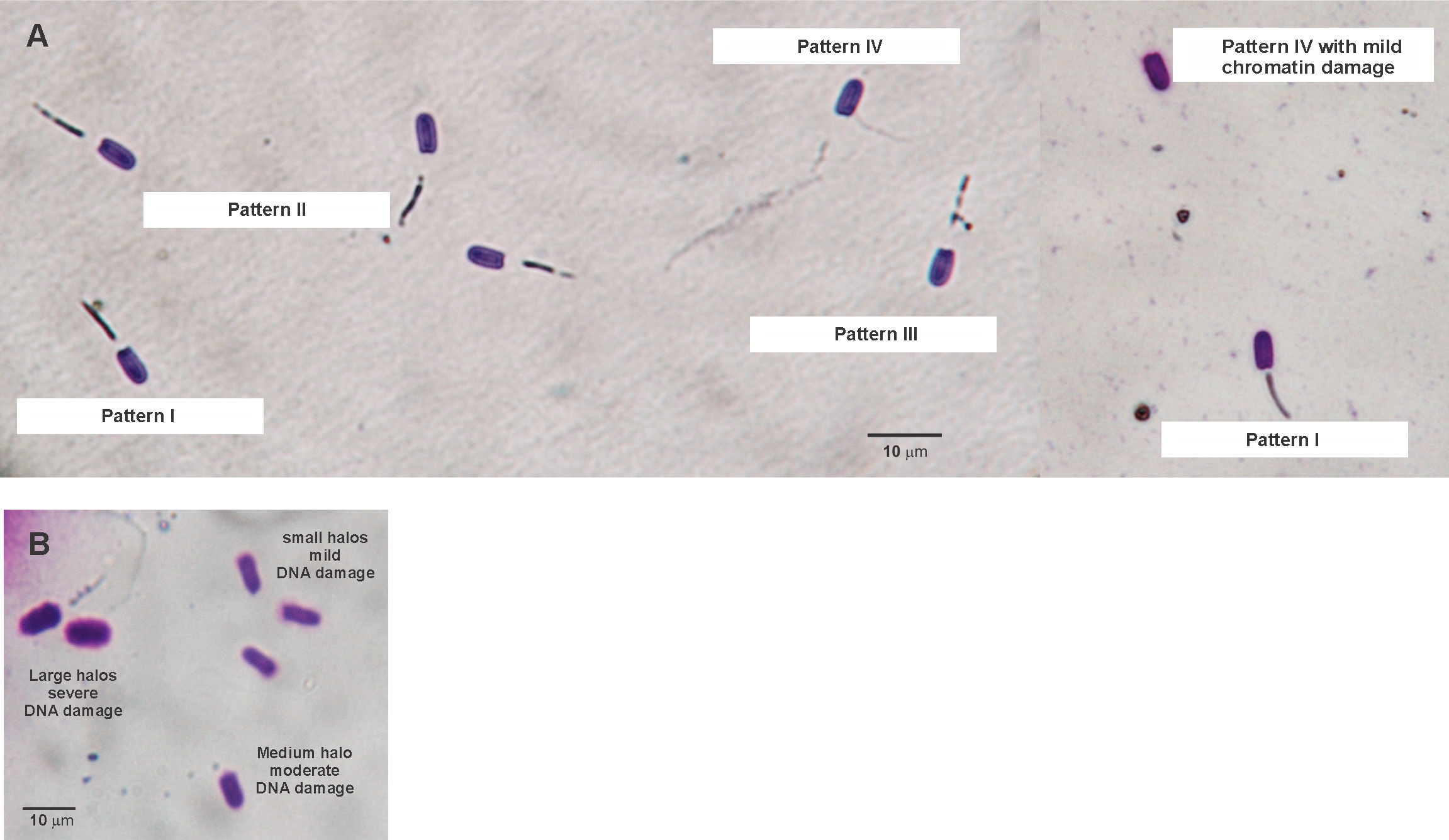

Supplement: Supplementary Figure 1 — Patterns of mid-piece staining and DNA integrity. (A) Microscope images of sperm showing different patterns of staining of the midpiece based on cytochrome c oxidase activity: pattern I, 100% of the midpiece stained, pattern II, more than 50% of the midpiece stained, pattern III, less than 50% of the midpiece stained, and pattern IV, no staining. (B) The brown color is product of DAB precipitation. (B) Micrograph showing different degrees of DNA damage based on Wright solution staining. Cells with large, medium or small halos of diffusion correspond to sperm nuclei containing fragmented DNA. Severe DNA damage was obtained by storing sperm at −80°C. Different grades of damage are seen on the micrograph (A). [file Image_1.TIF]
