# Supplementary material for: Effect of zinc on boar sperm liquid storage
Source: Front Vet Sci. 2023 Feb 2;10:1107929. doi: 10.3389/fvets.2023.1107929 (PMC9932539; doi:10.3389/fvets.2023.1107929)
Supplement: Supplementary Table 1 — Distribution and origin of boars used for AI trial. [file Table_1.docx]

| **Boar number** | **N° inseminated sows without zinc** | **N° inseminated sows with zinc** |
| --- | --- | --- |
| 57 | 96 | 40 |
| 56 | 41 | 15 |
| 52 | 7 | 7 |
| 53 | 4 | 4 |
| 42 | 3 | 3 |
| 40 | 3 | 3 |
| 43 | 3 | 3 |
| 51 | 3 | 3 |
| 27 | 2 | 2 |
| 95 | 1 | 1 |
| 38 | 1 | 1 |
| 50 | 1 | 1 |
| 54 | 1 | 1 |
| Total | 166 | 84 |

**Table S1.** The following boars were used in this study, numbered according to Medax company N°: 56, 57 (Sire Line PIC 337), and 27, 40, 42, 50, 51, 52, 53 (Sire Line PIC 415) from PIC®, Pig Improvement Company, Pasig City, Philippines; 38 (Large White) from La Botica Genética Porcina S.A., Buenos Aires, Argentina; 54 and 95 (Pietrain) from Cooperativa Marcos Juarez, Cordoba, Argentina; and 43 (Landrance) from Topigs Norsvin, Buenos Aires, Argentina.
