# Supplementary material for: Effect of zinc on boar sperm liquid storage
Source: Front Vet Sci. 2023 Feb 2;10:1107929. doi: 10.3389/fvets.2023.1107929 (PMC9932539; doi:10.3389/fvets.2023.1107929)
Supplement: Supplementary Table 2 — Comparison between two CASA systems with two different supports. [file Table_2.DOCX]

**Comparison between two CASA systems with two different supports.**

**Table S2**. Sperm motile parameters analyzed by the two computer-aided sperm analysis systems with two different supports (n = 9). Two microliters were loaded in ISAS® D4C20 chamber (Lot PR12-073-2) and 8 μl were loaded between slide and 18 mm x 18 mm coverslips

|  | Proiser + ISAS® D4C20 | IVOS I + Cover-Slide | *p* |
| --- | --- | --- | --- |
| Motile (%) | 90.67 ± 1.53 | 89.32 ± 6.25 | 0.9812 |
| VSL (μm/s) | 40.53 ± 1.23 | 42.16 ± 2.13 | 0.1010 |
| VCL (μm/s) | 113.01 ± 5.21 | 107.11 ± 6.02 | 0.0701 |
| VAP (μm/s) | 58.78 ± 2.56 | 59.35 ± 2.33 | 0.8317 |
| ALH (μm) | 5.33 ± 0.21 | 4.89 ± 0.63 | 0.6165 |
| STR (%) | 68.95 ± 4.23 | 71.03 ± 3.25 | 0.0596 |
| LIN (%) | 35.86 ± 2.03 | 39.36 ± 2.12 | 0.1195 |

VSL, straight line velocity; VCL, curvilinear velocity; VAP, average path velocity; ALH, amplitude of lateral head displacement; LIN, linearity; STR, straightness

**Statistics analysis**

Results are expressed as mean ± SD and analyzed with paired t-test. P < 0.05 was considered as a significant difference.
